# Total serum *N-*glycans associate with response to immune checkpoint inhibition therapy and survival in patients with advanced melanoma

**DOI:** 10.1186/s12885-023-10511-3

**Published:** 2023-02-18

**Authors:** Alessia Visconti, Niccolò Rossi, Helena Deriš, Karla A Lee, Maja Hanić, Irena Trbojević-Akmačić, Andrew M. Thomas, Laura A. Bolte, Johannes R. Björk, Jahlisa S. Hooiveld-Noeken, Ruth Board, Mark Harland, Julia Newton-Bishop, Mark Harries, Joseph J. Sacco, Paul Lorigan, Heather M. Shaw, Elisabeth G.E. de Vries, Rudolf S.N. Fehrmann, Rinse K. Weersma, Tim D. Spector, Paul Nathan, Geke A. P. Hospers, Peter Sasieni, Veronique Bataille, Gordan Lauc, Mario Falchi

**Affiliations:** 1 Department of Twins Research & Genetics Epidemiology, King’s College London, London, UK; 2 Genos Glycoscience Research Laboratory, Zagreb, Croatia; 3 CIBIO, University of Trento, Trento, Italy; 4 Department of Gastroenterology and Hepatology, University of Groningen and University Medical Center, Groningen, The Netherlands; 5 Department of Medical Oncology, University Medical Center Groningen, Groningen, The Netherlands; 6 Department of Oncology, Lancashire Teaching Hospitals NHS Trust, Chorley, UK; 7 Division of Haematology and Immunology, Institute of Medical Research at St. James’, University of Leeds, Leeds, UK; 8 Department of Medical Oncology, Guy’s and St Thomas’ NHS Foundation Trust, London, UK; 9 Liverpool Clatterbridge Cancer Centre, Liverpool, UK; 10 Department of Molecular and Clinical Cancer Medicine, University of Liverpool, Liverpool, UK; 11 The Christie NHS Foundation Trust, Manchester, UK; 12 Department of Medical Oncology, Mount Vernon Cancer Centre, Northwood, UK; 13 School of Cancer and Pharmaceutical Sciences, King’s College London, London, UK; 14 Department of Dermatology, Mount Vernon Cancer Centre, Northwood, UK; 15 Faculty of Pharmacy and Biochemistry, University of Zagreb, Zagreb, Croatia; 16 Department of Dermatology, West Herts NHS Trust, Herts, UK

**Keywords:** Melanoma, Immune checkpoint inhibitors, Total serum *N-*glycomics, Response, Survival

## Abstract

**Background:**

Immune checkpoint inhibitors (ICIs) have revolutionized the treatment of melanoma and other cancers. However, no reliable biomarker of survival or response has entered the clinic to identify those patients with melanoma who are most likely to benefit from ICIs. Glycosylation affects proteins and lipids’ structure and functions. Tumours are characterized by aberrant glycosylation which may contribute to their progression and hinder an effective antitumour immune response.

**Methods:**

We aim at identifying novel glyco-markers of response and survival by leveraging the *N-*glycome of total serum proteins collected in 88 ICI-naive patients with advanced melanoma from two European countries. Samples were collected before and during ICI treatment.

**Results:**

We observe that responders to ICIs present with a pre-treatment *N*-glycome profile significantly shifted towards higher abundancy of low-branched structures containing lower abundances of antennary fucose, and that this profile is positively associated with survival and a better predictor of response than clinical variables alone.

**Conclusion:**

While changes in serum protein glycosylation have been previously implicated in a pro-metastatic melanoma behaviour, we show here that they are also associated with response to ICI, opening new avenues for the stratification of patients and the design of adjunct therapies aiming at improving immune response.

**Supplementary Information:**

The online version contains supplementary material available at 10.1186/s12885-023-10511-3.

## Background

Glycosylation is a tightly regulated enzymatic process in which sugar chains are added to proteins or lipids to form glycoconjugates. This co- and post-translational modification generates different structures that modulate or mediate a wide variety of functions in health and disease. Cells present glycoconjugates on their outer surfaces, where they mediate cell signalling and communication, proliferation, differentiation, adhesion, and migration [1, 2]. Therefore, it is not surprising that tumours are characterized by aberrant glycosylation which may contribute to angiogenesis, invasion, and metastasis [3–5] and to the inhibition of an effective antitumour immune response [6].

Recently, multiple efforts have been spent in breaking the tumour glyco-code, with the double goal of identifying biomarkers of cancer development and progression, and devising novel therapeutic strategies via glycosylation manipulation [5–7]. These goals are also important to improve the treatment of cutaneous malignant melanoma, a malignancy with a high propensity to metastasise and whose incidence is rising faster than any other common cancer [8]: it is currently the fifth most common cancer in the UK and accounts for almost all skin-cancer related deaths [9]. Aberrant glycosylation, indeed, plays an important role in melanoma cells, where it promotes a pro-invasive and/or pro-metastatic phenotype [10, 11]. Several glycosyltransferases, enzymes responsible transferring specific glycans to proteins and lipids, showed abnormal abundances in metastatic cells, determining a corresponding abnormal amount of their enzymatic products [12–14]. Additionally, tumour hypersialylation promotes melanoma immune escape [15, 16].

The introduction of immune checkpoint inhibitors (ICIs) has revolutionized the treatment of advanced melanoma [17, 18], as well as other cancers [19], although a significant proportion of patients with melanoma are either non-responders or acquire resistance to ICI after an initial response [20]. Several, mostly tissue-based, biomarkers have been associated with response rate and survival to ICI in melanoma [21], but, currently, none have met the required tests of reliability and scalability. Thus, there is still no biomarker of survival or response used in the clinical setting for this cancer. Additionally, to overcome the limitations of tissue-based markers, the focus has shifted to the identification of peripheral blood-based, or *liquid*, biomarkers that could be easily detectable using non-invasive and repeatable methods.

Cancer cells have been shown to release aberrant glycan structures and glycoproteins in the bloodstream [5], where they can be detected and measured, particularly when tumour glycan epitopes involve tissue-specific proteins, such as aberrant circulating glycoforms of the prostate-specific antigen (PSA) in patients with prostate cancer [22]. In other cases, altered levels of circulating glycans may reflect both the aberrant expression and activity of glycosyltransferases and glycosidases in the tumour tissue, and the systemic response to cancer, which may involve acute-phase proteins being secreted by the liver and immunoglobulin G (IgG) glycoproteins being produced by B-cells [23].

Previous studies have shown that the measurement of serum glycan abundances and of the expression of enzymes involved in their synthesis and catabolism are promising approaches for early diagnosis of cancer and prediction of response to treatments and prognosis [24]. For instance, a study in a small cohort of 39 patients with advanced lung cancer demonstrated that individuals with decreased α1,3-fucosylated α1-acid glycoprotein (AGP) serum levels after treatment with nivolumab had better 2-year survival than those whose levels remained consistently high [25]. Additionally, preliminary results in a small cohort of 36 patients with metastatic melanoma receiving pembrolizumab alone or a combination of nivolumab and ipilimumab suggest that serum glycopeptides are promising biomarkers for predicting response to ICI treatment [26].

In this study, we hypothesise that the measurement of serum *N-*glycans assortment and abundance is a promising approach for identifying non-invasive biomarkers of response to ICI treatment and survival time in patients with advanced melanoma and carries the potential to improve immune responses by informing the development of adjunct therapy targets. To this end, we leverage pre-treatment and on-treatment serum *N-*glycan profiles in 88 patients with advanced melanoma receiving ICI to identify novel glyco-markers of response to therapy and overall and progression-free survival.

## Methods

### Study recruitment centres

The Predicting Response to Immunotherapy for Melanoma with Gut Microbiome and Metabolomics (PRIMM) study comprises two prospective observational cohort studies recruiting patients in parallel in the United Kingdom (PRIMM-UK) and the Netherlands (PRIMM-NL). PRIMM-UK (NCT03643289) recruited patients across several UK cancer centres, sponsored by East & North Hertfordshire NHS Trust with ethical approval from the South-Central Berkshire committee of the Research Ethics Services (RES) of the NHS. PRIMM-NL recruited Dutch patients from the COLIPI (METc number 2012/085, NCT02600143), POINTING (METc number 2018/350, NCT04193956), and OncoLifeS (METc number 2010/109) studies. All studies have been approved by the Medical Ethical Committee (in Dutch: Medisch Ethische Toetsingsingscommissie or METc) of the University Medical Center Groningen (UMCG) in the Netherlands. OncoLifeS information is available on the Netherlands Trial Register at https://www.trialregister.nl/trial/7839.

The University of Leeds recruited patients within a prospective cohort study named “Developing a Blood Test of Immunity in Illness: A study examining the peripheral blood transcriptome in patients with cancer, autoimmune disease, immunodeficiency or iatrogenic immune suppression” (REC Ref: 15/NW/0933, “Leeds” cohort).

Written informed consent was obtained from all patients.

### Study subjects

Patients with advanced cutaneous malignant melanoma were recruited from the three centres described above. Inclusion criteria were: *(i)* histologically or cytologically confirmed unresectable stage III or stage IV cutaneous melanoma, *(ii)* treatment with ICI (nivolumab, pembrolizumab, ipilimumab or a combination of ipilimumab and nivolumab) at recommended doses as first-line ICI, and *(iii)* 18 year of age or older.

### Clinical assessment

Collected clinical data used in this study include age at advanced melanoma diagnosis, sex, body mass index (BMI), *BRAF* mutation status, metastatic stage, lactate dehydrogenase (LDH) levels, Eastern Cooperative Oncology Group (ECOG) performance-status, prescribed ICI regimen, date of disease progression, and/or date of death. LDH levels were dichotomized according to the upper limit of normal for each centre.

Radiological evaluation, consisting of a computerized tomography (CT) scan of the thorax, abdomen and pelvis and magnetic resonance imaging (MRI) of the brain, was performed before starting immunotherapy. A small number of patients had positron emission tomography scans with a CT component. Follow-up radiological evaluation was performed every 10–14 weeks as long as the patient received systemic therapy. Additional CT and/or MRI scans were performed when there was suspicion of progression. If the first radiological evaluation after the start of therapy was inconclusive, a confirmatory scan was performed 4–12 weeks later.

Follow-up visits were scheduled every third week, and were grouped, according to the time from the start of treatment, into *(i)* early follow-up visits, *i.e.*, within 2–5 weeks after starting treatment and typically after the first ICI cycle, and *(ii)* late follow-up visits, *i.e.*, within 5–12 weeks from ICI start, and typically after the second or third ICI cycle. Follow-up visits within one week or after 12 weeks from ICI treatment initiation were discarded. A patient having pre-treatment and early follow-up samples taken more than 7 weeks apart was discarded from longitudinal analyses. When multiple visits were available within the same treatment window, the visit closest to the time window median (*i.e.*, 3 and 9 weeks for early and late follow-up visits, respectively) was used, and the other assessments were discarded.

Response to ICI was classified according to Response Evaluation Criteria in Solid Tumours (RECIST) v1.1 criteria [27]. Based on the radiographic response, patients with progressive disease (PD) on the first radiological evaluation that was confirmed on the next follow-up scan, patients with PD on the first radiological evaluation who were unable to complete a confirmation scan due to clinical progression or death, and patients who could not complete any radiological evaluation due to clinical progression or death were labelled non-responders. Patients that had a response (including complete response, partial response, or stable disease) at the first radiological evaluation that was confirmed on the next follow-up scan, and patients with PD on the first radiological evaluation but with a response at the second radiological evaluation (*i.e.*, late responders) were considered as responders. In late responders, progression at the first radiological evaluation was discarded.

Clinical endpoints included overall response rate (ORR, *i.e.*, whether the patient has a response to ICI), progression-free survival (PFS, *i.e.*, the time from the first dose of ICI to PD by RECIST v1.1, or death from any cause), and overall survival (OS, *i.e.*, the time from the first dose of ICI to death).

### Sample collection

Serum samples were collected up to four months before starting ICI (baseline samples) and during ICI therapy or within 21 days from the last ICI cycle (follow-up samples). Research nurses took venous blood samples at the respective hospitals in each country. Serum was obtained by centrifugation and stored at each site at − 80° C until shipping to King’s College London, UK, where they were aliquoted and sent to Genos Glycoscience Research Laboratory, Zagreb, Croatia, for the *N-*glycan analysis.

### ***N-***glycan analysis

Sample preparation and ultra-high-performance liquid chromatography (UHPLC) analysis of total serum *N-*glycome were performed as described elsewhere [28, 29]. Briefly, *N*-glycans were enzymatically released by PNGase F from 10 µL of serum samples, labelled with 2-aminobenzamide and cleaned up from the excess of reagents by hydrophilic interaction chromatography (HILIC) based solid-phase extraction (SPE). Fluorescently labelled *N-*glycans were separated by HILIC on a Waters BEH Glycan chromatography column (150 × 2.1 mm i.d., 1.7 μm) by UHPLC using an ACQUITY H-class instrument (Waters). Obtained serum *N-*glycan chromatograms were separated into 39 *N-*glycan peaks (Supplementary Fig. 1,Supplementary Table 1) whose composition had already been assigned [29].

To remove experimental variation and make *N*-glycan measurements comparable across samples, normalisation and batch correction were performed on UHPLC glycan data. More in detail, each directly measured glycan peak was first normalised by total chromatogram area. Then, the observed relative glycan abundances were log-transformed, and batch corrected using ComBat method [30] (R package sva, v 3.41.0).

Directly measured *N-*glycans with shared structural features were further summarized into 16 derived traits calculated from the normalized glycan data, as illustrated in Supplementary Table 2.

To investigate sample stratification due to recruitment centre and ICI therapy, we performed permutational multivariate analysis of variance (PERMANOVA; *adonis* R function, vegan package, v 2.5.7) using Euclidian distance on scaled directly measured glycomics data in pre-treatment samples, correcting for sex and age at advanced melanoma diagnosis, and performing 999 permutations.

### Power calculation

Power calculation was performed using the R package pwr (v 1.3.0). Specifically, we estimated the power to detect a Cohen’s conventional large effect size (*d*, that is the difference in *N*-glycan level means between responders and non-responders over the pooled standard deviation) of 0.8 [31], at an α-level of 0.05/N_eff_, where N_eff_ is the effective number of independent tests taking into account the strong correlation among *N*-glycans. N_eff_ was calculated using pre-treatment *N*-glycan relative abundances and the approach proposed by Li & Ji [32], resulting in N_eff_ = 20 and 6 for directly measured *N*-glycans and derived traits, respectively.

### Association between pre-treatment total serum ***N-***glycome and ORR

Outliers, defined as measurements deviating by more than three standard deviations from the mean of each *N-*glycan, were discarded from the association analyses. Relative abundances of directly measured *N-*glycans and of derived traits were inverse-normal transformed to ensure the normality of the data.

We explored the association between ORR and pre-treatment *N-*glycan levels using a linear model and correcting for sex, age at diagnosis of advanced melanoma, BMI, dichotomized LDH levels, and ECOG performance-status (*lm* R function, stats package, v 4.1.0). We considered an association significant when its *P* value passed a Bonferroni-derived threshold of 0.05/N_eff_.

Significant associations were further validated using permutation testing. Specifically, we generated 5,000 random datasets where labels indicating response status were randomly permuted. We then counted the number of times (T) the association *P* value in the simulated datasets was lower than 0.05/N_eff_, and used this number to estimate an empirical *P* value as *eP* = (T + 1)/5,001. We considered associations with measured *N-*glycans and derived traits confirmed when their *eP* was lower than 0.05/N_m_ and 0.05/N_d_, respectively, where N_m_ and N_d_ are the numbers of significant associations for measured *N-*glycans and derived traits, respectively.

Furthermore, we evaluated the power of serum *N*-glycans to predict ORR using three different generalized linear models, namely (*i*) a model including only clinical variables (*i.e.*, age, sex, BMI, metastatic stage, dichotomized LDH levels, and ECOG performance-status), (*ii*) a model including clinical variables and directly measured *N-*glycans associated with ORR, and (*iii*) a model including clinical variables and derived *N*-glycan traits associated with ORR. We used a leave-one-out cross-validation procedure (*train* R function, caret package, version 6.0.90) to evaluate models’ performances by means of the area under the receiver operating characteristic curve (AUC).

We used permutation testing to evaluate the contribution of the measured *N*-glycan and derived traits to models’ performance over clinical variables. We generated 1,000 datasets where the measured *N*-glycan/derived traits (but not age, sex, BMI, metastatic stage, dichotomized LDH levels, and ECOG performance-status) were randomly permuted as a single set, thus preserving their natural intra-subject correlation structure. For each random reshuffling of the dataset, we estimated the AUC of a generalised linear model to predict ORR using the same leave-one-out cross-validation procedure applied to the original dataset. An empirical *P* value (*eP*) was estimated as the probability of observing an AUC larger or equal than the one observed in the original dataset and considered significant when smaller than 0.05.

### Association between pre-treatment total serum ***N-***glycome and survival

The association between pre-treatment *N-*glycans and PFS and OS was assessed using the multivariate Cox proportional hazards regression analysis (*coxph* function, R package survival, v 3.2.13), including sex, age at diagnosis of advanced melanoma, BMI, dichotomized LDH levels, and ECOG performance-status as covariates. To assess violation of the proportional hazards assumption, we used the *cox.zph* function (survival R package) to obtain the global χ^2^ statistic. We considered the assumption met if the global χ^2^ *P* value was greater than 0.05. If the proportional hazards assumption was not met, the association was assessed in the short- (< 180 days), medium- (between 180 and 360 days) and long- (> 360 days) term.

The significance of the association was calculated using a formal likelihood-ratio test comparing the likelihood of the alternative model described above to the likelihood of a null model where the effect of *N-*glycans was constrained to zero. Models which failed to converge, likely due to the low number of events within the studied term, were discarded. We considered an association significant when its *P* value passed a Bonferroni-derived threshold of 0.05/N_eff_.

Significant associations were further validated using permutation testing. Specifically, we calculated the linear predictor for the null model and used the fitted values to divide our sample into quartiles. Then, we generated 5,000 random datasets where the *N-*glycans’ relative abundances were randomly permuted within these quartiles, thus preserving the relationship between the covariates and both the survival time and the *N-*glycans’ relative abundances. Next, we counted the number of times (T) the association in the random datasets was significant according to the criteria above and used this number to estimate an empirical *P* value as *eP* = (T + 1)/5,001. We considered associations with directly measured *N-*glycans and derived traits confirmed when their *eP* was lower than 0.05/N_m_ and 0.05/N_d_, respectively, where N_m_ and N_d_ are the numbers of significant associations for directly measured *N-*glycans and derived traits, respectively (for PFS: N_m_=4, N_d_=7; for OS: N_m_=5, N_d_=8).

To graphically present our associations, Kaplan-Meier curves were constructed to compare PFS and OS between low, medium, and high *N-*glycan relative abundances groups, defined by tertile cut-off points per each *N-*glycan, across samples (*ggsurvplot* function, R package survminer, v 0.4.9).

### ***N-***glycomics shift after ICI and its association with ORR

To explore changes in serum *N-*glycans due to ICI treatment, we compared their pre-treatment and on-treatment relative abundances in the combined set of responders and non-responders at both early and late follow-up windows using a paired two-sided Wilcoxon test. We considered an association significant when its *P* value passed a Bonferroni-derived threshold of 0.05/N_eff_. Significant associations were further validated via permutation testing using the procedure described above.

The null distribution of the test statistics was simulated by assessing the longitudinal variation of *N*-glycan levels in 1,000 random datasets where pre-treatment and on-treatment *N*-glycan values were swapped for a random number of patients, under the hypothesis that if ICI-treatment does not have any effect the two measurements are interchangeable. For *N*-glycans showing statistically significant difference between baseline and follow-up samples, we explored the association between their shift (obtained by subtracting measurements taken at follow-up from those taken at baseline) and ORR using a linear model and correcting for sex, age at diagnosis of advanced melanoma, BMI, dichotomized LDH levels, and ECOG performance-status (*lm* R function, stats package, v 4.1.0). We considered the association significant when its *P* value was lower than a Bonferroni-derived threshold of 0.05/2 = 0.0125.

## Results

### Patients’ characteristics

This study included 88 previously ICI-naive patients with advanced melanoma who received their first cycle of ICI between May 2017 and January 2020 from the PRIMM-UK (*N* = 34), PRIMM-NL (*N* = 40), and Leeds (*N* = 14) recruitment centres (Table 1, Methods). They were classified into 49 responders, *i.e.*, patients who had a complete or partial response or presented with sustained stable disease, as assessed by RECIST v1.1 at the first and/or second radiological evaluation, and 39 non-responders (Table 1, Methods).

**Table 1:** Patient characteristics. Categorical variables are presented as number (percentage). Continuous variables are presented as mean ± standard deviation. Differences between recruitment centres were assessed using Fisher’s exact test, for categorical variables, and one-way ANOVA, for continuous variables. Follow-up samples were collected 2–12 weeks after ICI initiation

	All cohorts	Leeds	PRIMM-NL	PRIMM-UK	*P*
**N (pre-treatment)**	88	14	40	34	-
**N (follow-up)**	66	11	28	27	-
**Sex**					
* Male*	57 (64.8%)	9 (64.3%)	23 (57.5%)	25 (73.5%)	0.35
* Female*	31 (35.2%)	5 (35.7%)	17 (42.5%)	9 (26.5%)	
**Age (years)**	60.5 ± 15.0	57.4 ± 14.6	59.4 ± 12.7	66.0 ± 16.8	0.08
**BMI (kg/m**^**2**^**)**	28.0 ± 5.4	27.6 ± 5.2	27.6 ± 5.5	28.5 ± 5.4	0.72
***BRAF*** **mutant**	40 (45.5%)	8 (61.5%)	23 (57.5%)	9 (26.5%)	0.01
**LDH (≤ULN)**	58 (65.9%)	5 (35.7%)	30 (75.0%)	23 (67.6%)	0.03
**Metastatic stage**					
* Stage III unresectable*	2 (2.3%)	1 (7.1%)	1 (2.5%)	0 (0.0%)	2.2 × 10^− 4^
* M1a*	14 (15.9%)	2 (14.3%)	2 (5.0%)	10 (29.4%)	
* M1b*	17 (19.3%)	3 (21.4%)	4 (10.0%)	10 (29.4%)	
* M1c*	32 (36.4%)	6 (42.9%)	14 (35.0%)	12 (35.3%)	
* M1d*	23 (26.1%)	2 (14.3%)	19 (47.5%)	2 (5.9%)	
**ECOG performance status**					
0	47 (53.4%)	12 (85.7%)	25 (62.5%)	10 (29.4%)	5.9 × 10^− 3^
1	31 (35.2%)	2 (14.3%)	10 (25.0%)	19 (55.9%)	
2	8 (9.1%)	0 (0.0%)	4 (10.0%)	4 (11.8%)	
3	2 (2.3%)	0 (0.0%)	1 (2.5%)	1 (2.9%)	
**ICI therapy**					
* Ipilimumab*	1 (1.1%)	0 (0.0%)	1 (2.5%)	0 (0.0%)	0.02
* Pembrolizumab*	20 (22.7%)	2 (14.3%)	7 (17.5%)	11 (32.4%)	
* Nivolumab*	30 (34.1%)	5 (37.5%)	20 (50.0%)	5 (14.7%)	
* Ipilimumab + Nivolumab*	37 (42.0%)	7 (50.0%)	12 (30.0%)	18 (52.9%)	
** ORR**	49 (55.7%)	9 (64.3%)	21 (52.5%)	19 (55.9%)	0.77
** PFS (months)**	5.6 ± 5.3	7.0 ± 9.3	5.3 ± 4.3	5.7 ± 5.0	0.74
** OS (months)**	9.0 ± 6.6	3.8 ± 1.4	11.0 ± 7.1	7.8 ± 5.7	0.08

*BMI* Body mass index, *LDH* Lactate dehydrogenase, *ULN* Upper limit of normal, *ECOG* Eastern cooperative oncology group performance-status, *ICI* Immune checkpoint inhibitor, *ORR* Overall response rate, *OS* Overall survival, *PFS* Progression-free survival

Patients had an average age of 60.5 ± 15.0 years at diagnosis of advanced melanoma (range: 25–94) and were predominantly males (65%). Patients’ sex, age at diagnosis of advanced melanoma, and BMI were similar between recruitment centres (*P* > 0.05, Fisher’s exact test). Despite patients’ *BRAF* mutation status, metastatic stage, dichotomized LDH levels, ECOG performance-status, and prescribed ICI regimen differing across recruitment centres (*P* < 0.03, Fisher’s exact test), we observed comparable ORR, PFS, and OS (*P* > 0.05, Fisher’s exact test or analysis of variance [ANOVA]).

ORR was not associated with patients’ age, *BRAF* mutation status, metastatic stage, dichotomized LDH levels, ECOG performance-status, prescribed ICI treatment, or BMI (after correcting for age and sex; *P* > 0.05). Conversely, ORR was associated with patients’ sex, with females having higher odds of positive response to ICI compared to males (odds ratio [OR] = 4.31, 95% confidence interval [CI] = 1.50–13.9, *P* = 3.3 × 10^− 3^, Fisher’s exact test).

The average PFS was 6 months, with 35, 11, and 6 patients progressing within 6, 12, or after 12 months, respectively (Table 1, Supplementary Fig. 2). The average OS was 9 months, with 12, 13, and 8 deaths recorded within 6, 12, or after 12 months, respectively (Table 1, Supplementary Fig. 2). PFS and OS durations did not differ by sex, *BRAF* mutation status, metastatic stage, ECOG performance-status, prescribed ICI regimen (*P* > 0.05, ANOVA or Wilcoxon’s test), or BMI (after correcting for age and sex; *P* > 0.05). OS was negatively associated with age at diagnosis of advanced melanoma (β = -5.0, SE = 1.9, *P* = 0.01) and with dichotomized LDH levels (*P* = 0.02, Wilcoxon’s test).

Sixty-six patients had at least one serum sample collected at follow-up during ICI treatment. Follow-up samples were divided, according to the time difference between ICI initiation and sample collection, into 54 early (from 2 to 5 weeks) and 36 late (from 5 to 12 weeks) follow-up samples, with 24 patients having one sample collected at both early and late follow-up windows; Methods**, **Supplementary Fig. 2). The median number of days between treatment initiation and follow-up sample collection was 21 and 43 days for the early and late treatment window, respectively.

### **Pre-treatment total serum*N-*glycome composition associates with response to ICI**

*N-*glycosylation is the most common type of protein glycosylation [33] and occurs when a sugar chain is transferred to an asparagine residue in a sequence Asn-X-Ser/Thr, where X is any amino acid except proline. Here, we directly measured 39 serum *N-*glycans (Supplementary Fig. 1 and Supplementary Table 1) which were further summarised into 16 derived *N-*glycan traits (Supplementary Table 2). Derived traits consist of structurally similar *N-*glycans and therefore average specific glycosylation features (e.g., galactosylation, fucosylation, sialylation) across the different measured *N-*glycan structures, thus providing a more composite and robust measure of the enzymatic activities responsible for the glycans’ biosynthesis than directly measured *N-*glycans.

We observed no significant differences in pre-treatment *N*-glycan levels according to recruitment centres, patients’ *BRAF* mutation status, metastatic stage, or ICI treatment (permutational multivariate analysis of variance [PERMANOVA] *P* > 0.05), after correcting for age and sex. Conversely, we observed significant differences in pre-treatment *N*-glycan levels according to dichotomized LDH levels and ECOG performance-status, as well as in directly measured *N-*glycans according to BMI (PERMANOVA *P* < 0.05). Therefore, we investigated the association between pre-treatment *N-*glycans and ORR using a linear model and correcting for age, sex, dichotomized LDH levels, ECOG performance status, and BMI in 49 responders and 39 non-responders (Methods). In this sample, we had 72% and 84% power to detect a difference with a Cohen’s *d* > 0.8 between responders and non-responders at a Bonferroni-derived threshold of 0.05/20 = 2.5 × 10^− 3^ and 0.05/6 = 8.3 × 10^− 3^, for measured *N-*glycans and derived traits, respectively (Methods).

**Fig. 1:**
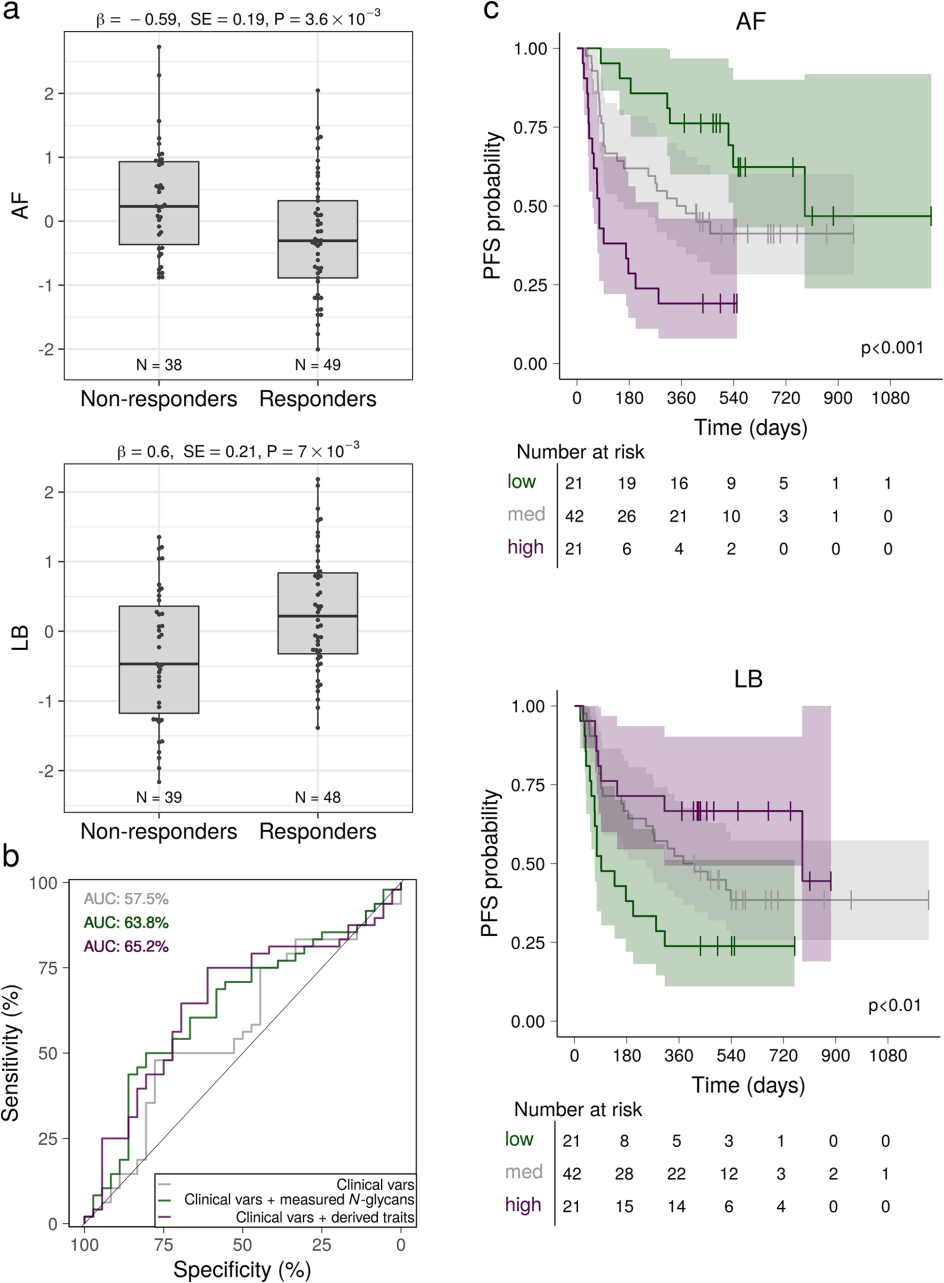
(**a**) Significant associations between serum derived *N-*glycan traits and ORR in 88 patients with advanced melanoma. Inverse-normalised age- and sex-corrected values are plotted, and each boxplot reports effect size (β), standard error (SE), and *P* value (P) of the linear regression analysis (Wilcoxon’s tests *P* values = 2.5 × 10^− 4^ and 1.2 × 10^− 2^ for uncorrected relative abundances of *N*-glycans containing antennary fucose and low-branched mono- and biantennary *N*-glycans, respectively). **b** Power of clinical variables and pre-treatment total serum ***N-***glycans in predicting ORR. The grey line represents the performance obtained when ORR is predicted by a model including only clinical variables, *i.e.*, age, sex, BMI, metastatic stage, dichotomized LDH levels, and ECOG performance-status. The green and magenta lines represent the performance resulting when the measured *N-*glycan (GP6) and derived traits (*N*-glycans containing antennary fucose and low-branched mono- and biantennary *N*-glycans) associated with ORR are included in the model, respectively. **c** Kaplan-Meier overall survival curves in 88 patients with advanced melanoma (derived *N*-glycan traits). Patients are divided into low (< 1st interquartile; green line), medium (> 1st interquartile and < 3rd interquartile; grey line), and high (> 3rd quartile; magenta line) according to pre-treatment serum *N-*glycan relative abundances. Log-rank test *P* values are shown. Abbreviations: *AF*: *N*-glycans containing antennary fucose bond by α1,3 linkage to the antenna GlcNAc residues, *LB*: low-branched mono- and biantennary *N*-glycans

We identified significant associations between ORR and two derived traits describing *N*-glycans containing antennary fucose (*i.e.*, bond by α1,3 linkage to the antenna GlcNAc residues) and low-branched mono- and biantennary N-glycans. Specifically, pre-treatment levels of *N*-glycans containing antennary fucose were lower in responders compared to non-responders (β = -0.59, SE = 0.19, *P* = 3.6 × 10^− 3^), whereas low-branched mono- and biantennary *N*-glycans were higher in responders (β = 0.60, SE = 0.21, *P* = 7.0 × 10^− 3^; Fig. 1a, Supplementary Table 3), with the two derived traits being anticorrelated (Supplementary Fig. 3). In addition, pre-treatment levels of the measured *N*-glycan GP6 (FA2 [6] BG1) were higher in responders compared to non-responders (β = 0.67, SE = 0.21, *P* = 1.8 × 10^− 3^; Supplementary Fig. 4, Supplementary Table 3).

The AUCs for response prediction were 63.8% and 65.2%, respectively, when the measured *N*-glycan and the derived traits associated with ORR were included as predictors in the regression model, together with age, sex, BMI, metastatic stage, dichotomized LDH levels, and ECOG performance-status (Fig. 1b). Permutation analysis showed that both the measured *N*-glycan and derived traits have a relevant contribution over clinical variables to discriminate between responders and non-responders, as the AUC dropped to 57.5% when they were excluded from the set of predictors (*eP* = 1.40 × 10^− 2^ and *eP* = 4.00 × 10^− 3^, respectively).

### Pre-treatment total serum ***N-***glycome composition associates with PFS and OS

We investigated whether pre-treatment serum *N*-glycans are associated with PFS and OS using a Cox proportional hazards regression analysis. Age, sex, BMI, dichotomized LDH levels, and ECOG performance-status were included as covariates, and significant associations were further confirmed via permutation testing (Methods). The two derived traits which were associated with ORR at baseline, namely *N*-glycans containing antennary fucose and low-branched mono- and biantennary *N*-glycans, were associated also with PFS. *N*-glycans containing antennary fucose were associated with reduced PFS (hazard rate (HR) = 1.91, 95% CI = 0.1.35–2.70, *P*_LRT _= 4.7 × 10^-4^), whereas low-branched mono- and biantennary *N*-glycans were associated with improved PFS (HR = 0.55, 95% CI = 0.38–0.79, *P*_LRT _= 9.4 × 10-4; Fig. 1c, Supplementary Table 4). Additionally, we identified three directly measured *N*-glycans associated with the hazard rate of progression: GP6, also associated with ORR, showed a beneficial effect on PFS (HR = 0.62, 95% CI = 0.45–0.84, *P*_LRT _= 1.4 × 10^-3^), whereas GP27 (A3F1G3S2/A3G3S3) and GP33 (A2BG2) were associated with shorter PFS (HR = 1.87, 95% CI = 1.33–2.63, *P*_LRT _= 4.5 × 10^-4^ and HR = 1.97, 95% CI = 1.36–2.84, *P*_LRT _= 4.1 × 10^-4^, respectively; Supplementary Fig. 5, Supplementary Table 4).

When testing the association between pre-treatment serum *N*-glycans and OS, we observed a time-varying, non-linear effect of age on mortality risk (Supplementary Fig. 6), resulting in a violation of the proportional hazard assumption of the Cox model. Thus, we partitioned the survival time axis into three time intervals based on events distribution and assessed the association between pre-treatment *N*-glycans and OS in the short- (< 180 days), medium- (180–360 days) and long- (> 360 days) term. Two directly measured *N*-glycans were associated with increased rate of death in the short- and medium-term: GP27 (short-term: HR = 3.64, 95% CI = 1.51–8.76, *P*_LRT _= 1.9 × 10^-3^; medium-term: HR = 3.45, 95% CI = 1.64–7.25, *P*_LRT _= 7.0 × 10^-4^) and GP33 (short-term: HR = 3.46, 95% CI = 1.52–7.88, *P*_LRT _= 1.9 × 10^-3^; medium-term: HR = 4.33, 95% CI = 1.82–10.31, *P*_LRT _= 3.5 × 10^-4^; Supplementary Fig. 7, Supplementary Table 5). Additionally, the directly measured *N*-glycan GP39 (A4F1G4S4/A4F2G4S4) was associated with reduced survival in the medium-term (HR = 3.57, 95% CI = 1.73–7.37, *P*_LRT _= 8.8 × 10^-4^; Supplementary Fig. 7, Supplementary Table 5).

Derived traits associated with ORR were nominally associated with OS in the short- and/or medium-term, with consistent direction of association to those observed for ORR and PFS: pre-treatment serum levels of *N*-glycans containing antennary fucose were negative prognostic markers of survival (short-term: HR = 3.28, 95% CI = 1.55–6.94, *P*_LRT _= 1.8 × 10^− 3^; medium-term: HR = 4.14, 95% CI = 1.80–9.49, *P*_LRT _= 3.7 × 10^−4^), whereas a favourable association with survival time was observed for low-branched mono- and biantennary *N*-glycans (medium-term: HR = 0.33, 95% CI = 0.15–0.71, *P*_LRT _= 2.4 × 10^− 3^) and GP6 (short-term: HR = 0.33, 95% CI = 0.12–0.92, *P*_LRT _= 1.6 × 10^− 2^; medium-term: HR = 0.53, 95% CI = 0.28–1.01, *P*_LRT _= 3.9 × 10^− 2^; Supplementary Table 5).

### A shift in serum proteins ***N-***glycosylation is observed after ICI treatment

We investigated whether ICI treatment induces a shift in pre-treatment serum *N*-glycans using data from 66 patients, including both responders and non-responders (Methods). While no significant differences were identified for derived traits, we observed two directly measured *N*-glycans showing a significant shift from baseline at the early follow-up window (*N* = 54, N_Responders _= 34, N_Non−responders _= 20): GP8 (A2G2; median_difference _= 0.035, paired Wilcoxon’s test *P* = 2.2 × 10^− 3^) and GP22 (FA2G2S2; median_difference _= -0.098, *P* = 2.2 × 10^− 3^; Supplementary Table 6), with GP22 showing a nominally significant decrease from baseline also at the late follow-up window (*N* = 36; N_Responders _= 26, N_Non−responder _= 10; median_difference _= -0.215, *P* = 0.01; Supplementary Table 6).

Driven by these results, we tested whether the longitudinal variation in the relative abundances of GP8 and GP22 at the early follow-up window was associated with ORR after correcting for age, sex, BMI, dichotomized LDH levels, and ECOG performance-status, but did not identify any significant result (*P* > 0.05, Methods). In this sample, we had 70% power to detect a difference in *N-*glycans longitudinal variation between responders and non-responders with a Cohen’s *d* > 0.8 at a Bonferroni-derived threshold of 0.05/2 = 0.025 (Methods). This suggests that, although ICI treatment could cause a similar shift of these *N-*glycans in both responders and non-responders, a larger sample size might be needed to identify a significant differential effect on response.

## Discussion

Aberrant glycosylation of melanoma cell lines has been previously implicated in pro-invasive and/or pro-metastatic melanoma behaviour [12, 13] and immune escape [17, 18]. However, *N-*glycosylation changes of total serum proteins as a proxy for melanoma progression and/or treatment response have not been studied in much depth yet. Here, we observe that the pre-treatment total serum *N*-glycome of patients with advanced melanoma who responded to ICI treatment is significantly shifted towards low-branched mono- and biantennary structures containing lower abundances of antennary fucose, and these changes are also associated with survival.

Several small studies have previously observed an elevated abundance of antennary fucosylation in the total plasma *N-*glycome in patients with colorectal cancer [34] as well as in haptoglobin of patients with pancreatic [35], hepatic [36], and prostatic [37] cancers compared to controls, highlighting the importance of fucosylation across several malignancies [38]. One of the main sources of (high-branched) antennary fucosylated *N-*glycans in serum is AGP [39], an acute-phase protein primarily synthesized in hepatocytes [40] and extrahepatically by carcinoma cells, especially in its α1,3-antennary fucosylated form [41] that we observed here to be inversely associated with ICI response. In line with our observations, lower abundance of circulating antennary fucosylated AGP was a good predictor of response and increased survival after ICI treatment with nivolumab in a smaller cohort of 39 patients with lung cancer [25]. Another small study examined *N-*glycosylation of circulating AGP in 18 patients with advanced melanoma and revealed an increased abundance of high-branched fucosylated glycans compared to the 19 healthy controls [42]. Taken together, these observations suggest that AGP glycosylation may have a role in melanoma progression as well as in ICI response and that additional studies are needed to confirm its role and understand the underlying mechanisms.

We also observed significantly higher relative abundance of mono- and bi-antennary *N-*glycans in responders compared to non-responders. A shift to more high-branched *N-*linked glycans is a well-known hallmark of cancer [1, 3], and is associated with tumour progression and metastatic development in melanoma, breast and colorectal cancer, amongst other malignancies [12, 43–46]. Branched *N*-glycans on tumour cells can impede antitumour immune attack and the removal of β1,6-GlcNAc-branched *N-*glycans in gastrointestinal cancer cells promotes immunostimulation by exposing immunogenic mannose-enriched glycans [46].

Among the measured *N-*glycans, we also observed that the relative abundance of GP6 (FA2B [6] G1), predominantly originating from IgG, was increased in responders compared to non-responders. An altered level of this glycan has been observed in endometrial cancer [47].

We found that the combination of pre-treatment serum *N*-glycan levels and clinical variables significantly increased response prediction accuracy compared to clinical variables alone, reaching an AUC of 63.8% and 65.2%, for measured *N*-glycans and derived traits, respectively. While these moderate AUC values warrant further investigation and optimization in larger datasets, they suggest that the total serum *N*-glycome may provide leads for non-invasive biomarkers of ICI efficacy for clinical use.

As expected, the two derived traits and the measured *N-*glycan associated with ICI response were also significantly associated with improved PFS, with concordant directions of effect.

ICI treatment was associated with higher GP8 and lower GP22 relative abundances at 2–5 weeks from the start of the treatment, both returning to pre-treatment levels after further 7 weeks. While these shifts were not significantly different between responders and non-responders, we cannot exclude that differences may be uncovered using a larger sample size.

This is an observational study and cannot prove a causal implication of *N-*glycans in ICI response and survival. It has also a relatively small sample size and lacks an external validation sample. However, it is one of the largest to date investigating the role of the total serum *N-*glycome in ICI response in advanced melanoma, moving forward from previous studies which focused almost exclusively on melanoma cell lines, and paving the way for the identification of liquid biomarkers that can be collected using non-invasive and repeatable methods. Moreover, here, we simultaneously investigated 39 total serum *N*-glycans and the assessed additional 16 derived traits summarising specific *N*-glycosylation features that are robust proxies for the enzymatic activities involved in the glycans’ biosynthesis, representing an improvement upon most previous studies that predominantly focused on single glycans.

## Conclusion

In summary, while future studies are needed to better understand the role and clinical applicability of the serum *N*-glycome in ICI response prediction in advanced melanoma, we show here that serum samples can be used for non-invasive investigation and identification of *N*-glycomic signatures of clinical response to ICI, thus representing one step forward toward personalised strategies for maximising ICI therapy success.

## Supplementary Information

**Additional file 1:** **Figure** **1.** Representative chromatogram of total human plasma/serum *N-*glycome separated byHILIC-UPLC into 39 *N-*glycan peaks (GP1-GP39). **Figure 2.** Swimmer plot for the 88 patients with advanced melanoma included in this study. **Figure 3.** Pearson's correlation among the measured *N-*glycan and derived traits associated with overall response rate in 88 patients with advanced melanoma. **Figure 4.** Significant association between directly measured serum *N*-glycans and overall response rate. **Figure 5.** Kaplan-Meier progression-free survival curves in 88 patients with advanced melanoma (directly measured *N-*glycans). **Figure 6.** Diagnostic plots for Cox proportional hazards regression model of survival time on sex, age at diagnosis of advanced melanoma, BMI, dichotomized LDH levels, and ECOG performance-status. **Figure 7.** Kaplan-Meier overall survival curves in 88 patients with advanced melanoma (directly measured *N-*glycans).

**Additional file 2: Table 1.**  Description of total serum *N*-glycan UHPLC measured peaks. **Table 2.**  Description of total serum derived traits. **Table 3.** Summary statistics for the associations between *N*-glycan traits and response to ICI treatment. **Table 4.** Summary statistics for the associations between *N*-glycan traits and progression-free survival. **Table 5.** Summary statistics for the associations between *N*-glycan traits and overall survival. **Table 6.** Summary statistics for the *N*-glycans shift at follow up with respect to the pre-treatment relative abundances.

## Data Availability

Data generated during the study are included in the article and/or Supplementary Material. Anonymized raw data on study participants are available to *bona fide* researchers under managed access due to governance and ethical constraints, and should be requested *via* contact with the corresponding authors.

## Abbreviations

AGP: α1-acid glycoprotein
AUC: area under the receiver operating characteristic curve (AUC)
BMI: body mass index
CI: confidence interval
CT: computerized tomography
ECOG: Eastern Cooperative Oncology Group
eP: empirical *P* value
GP: glycan peak
HILIC: hydrophilic interaction chromatography
HR: hazard rate
ICI: immune checkpoint inhibitor
IgG: Immunoglobulin G
LDH: lactate dehydrogenase
MRI: magnetic resonance imaging
OR: odds ratio
ORR: overall response rate
OS: overall survival
PD: progressive disease
PFS: progression-free survival
PSA: prostate-specific antigen
RECIST: Response Evaluation Criteria in Solid Tumours
UHPLC: ultra-high-performance liquid chromatography
