# Supplementary material for: Total serum N-glycans associate with response to immune checkpoint inhibition therapy and survival in patients with advanced melanoma
Source: BMC Cancer. 2023 Feb 18;23:166. doi: 10.1186/s12885-023-10511-3 (PMC9938582; doi:10.1186/s12885-023-10511-3)
Supplement: Supplementary file 1 — Additional file 1: Figure 1. Representative chromatogram of total human plasma/serum N-glycome separated byHILIC-UPLC into 39 N-glycan peaks (GP1-GP39). Figure 2. Swimmer plot for the 88 patients with advanced melanoma included in this study. Figure 3. Pearson's correlation among the measured N-glycan and derived traits associated with overall response rate in 88 patients with advanced melanoma. Figure 4. Significant association between directly measured serum N-glycans and overall response rate. Figure 5. Kaplan-Meier progression-free survival curves in 88 patients with advanced melanoma (directly measured N-glycans). Figure 6. Diagnostic plots for Cox proportional hazards regression model of survival time on sex, age at diagnosis of advanced melanoma, BMI, dichotomized LDH levels, and ECOG performance-status. Figure 7. Kaplan-Meier overall survival curves in 88 patients with advanced melanoma (directly measured N-glycans). [file 12885_2023_10511_MOESM1_ESM.pdf]

Supplementary Figures for

Total serum N-glycans associate with response to immune checkpoint inhibition therapy and survival in patients with advanced melanoma

Visconti et al

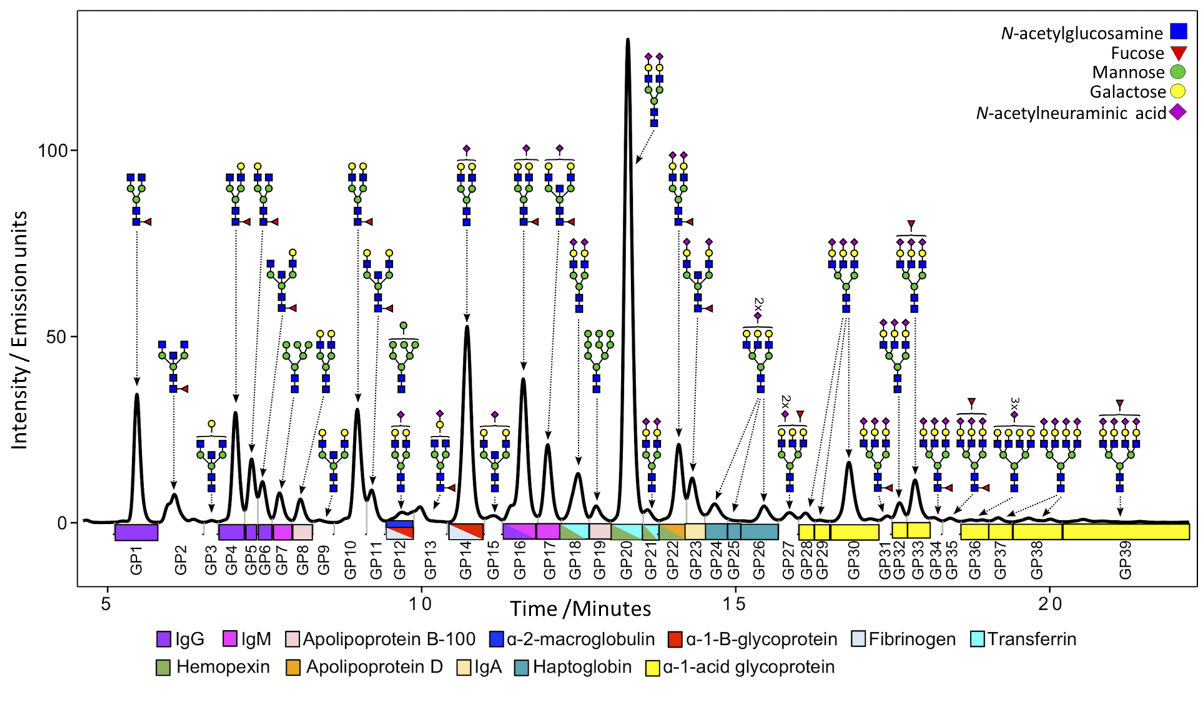

Supplementary Figure 1. Representative chromatogram of total human plasma/serum *N*-glycome separated by HILIC-UPLC into 39 *N*-glycan peaks (GP1-GP39). For each peak, major *N*-glycan structures confirmed by MS/MS analysis are shown. The colour of the boxes under chromatographic peaks represents the main glycoprotein sources of the major *N*-glycan structures contained in the peak according to [doi:10.1007/s10719-015-9626-2]. Reprinted (adapted) with permission from Zaytseva O.O., *et al.,* Heritability of Human Plasma *N*-Glycome*, J. Proteome Res.* 2020, 19, 1, 85–91 [doi:10.1021/acs.jproteome.9b00348]. Copyright 2020 American Chemical Society.

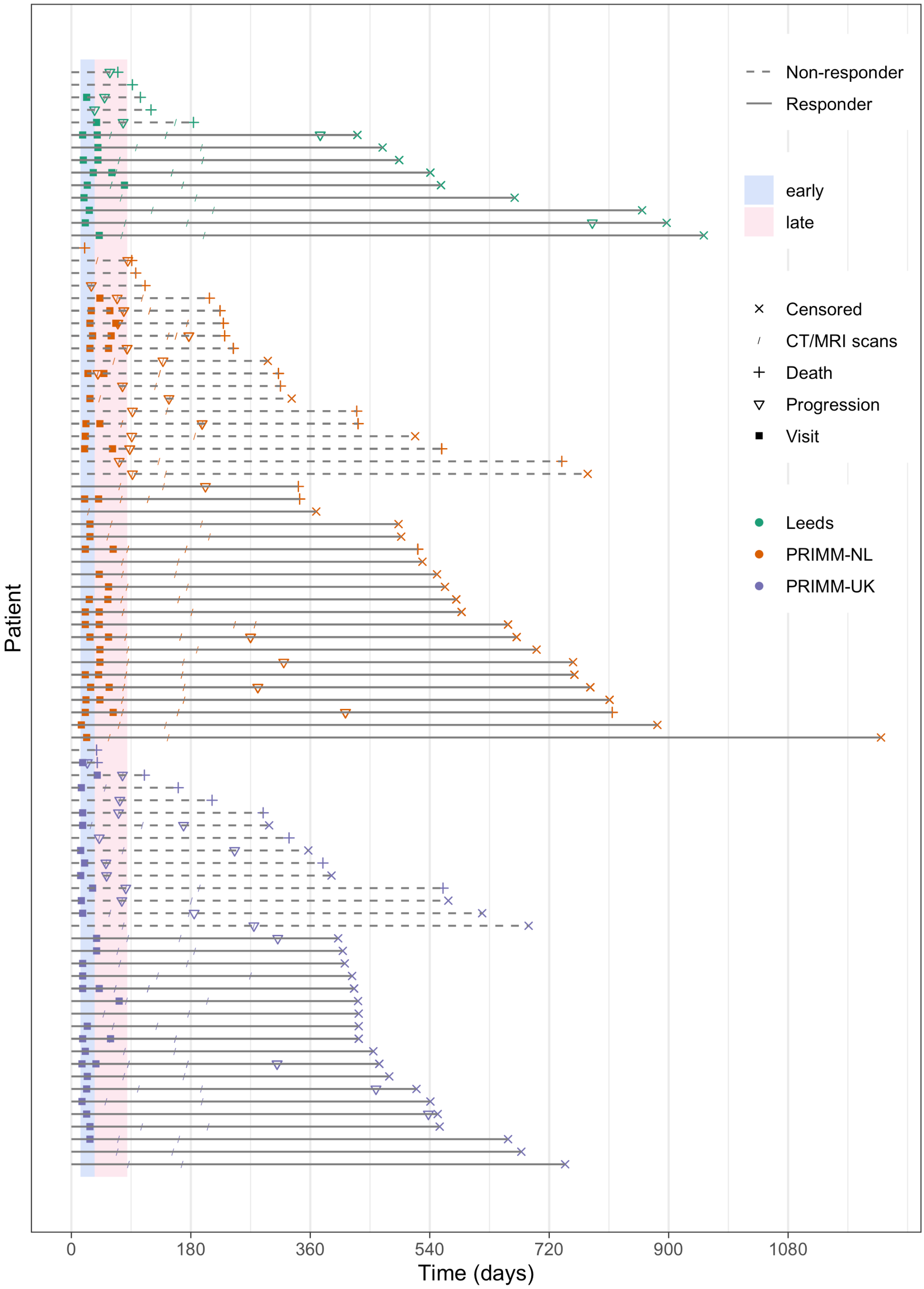

Supplementary Figure 2. Swimmer plot for the 88 patients with advanced melanoma included in this study. Day 0 indicates the day of ICI treatment initiation. Follow-up visits were available for 66 patients. Only visits carried out during the early (blue, 2 to 5 weeks from ICI treatment initiation) and late (red, 5 to 12 weeks from ICI treatment initiation) treatment window are included.

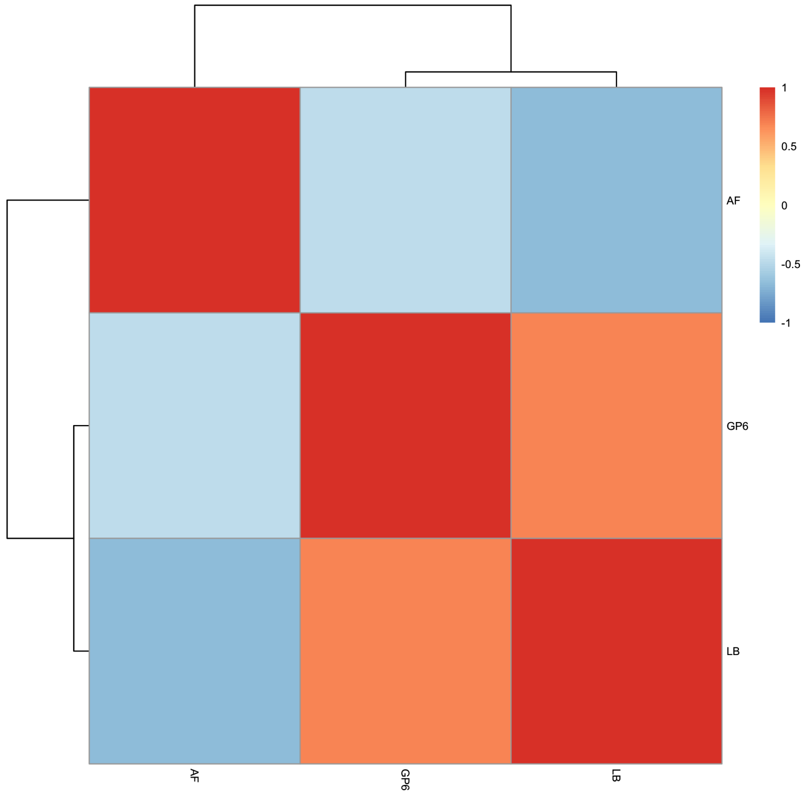

Supplementary Figure 3. Pearson's correlation among the measured *N*-glycan and derived traits associated with overall response rate in 88 patients with advanced melanoma. Structure for the measured *N*-glycan: GP6: FA2[6]BG1. Structures for the derived *N*-glycan trais: AF: *N*-glycans containing antennary fucose bond by α1,3 linkage to the GlcNAc residues, LB: low-branching mono- and biantennary *N*-glycans

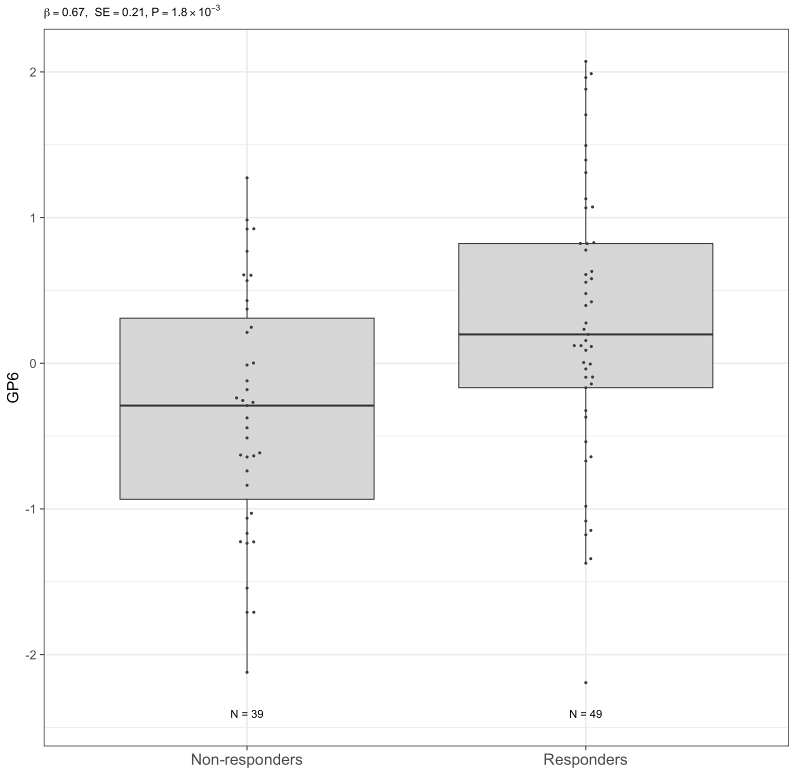

Supplementary Figure 4. Significant association between directly measured serum *N*-glycans and overall response rate. Inverse-normalised age- and sex-corrected values are plotted, and the boxplot reports effect size (β), standard error (SE), and *P* value (P) of the linear regression analysis. Structure for the measured *N*-glycan: GP6: FA2[6]BG1

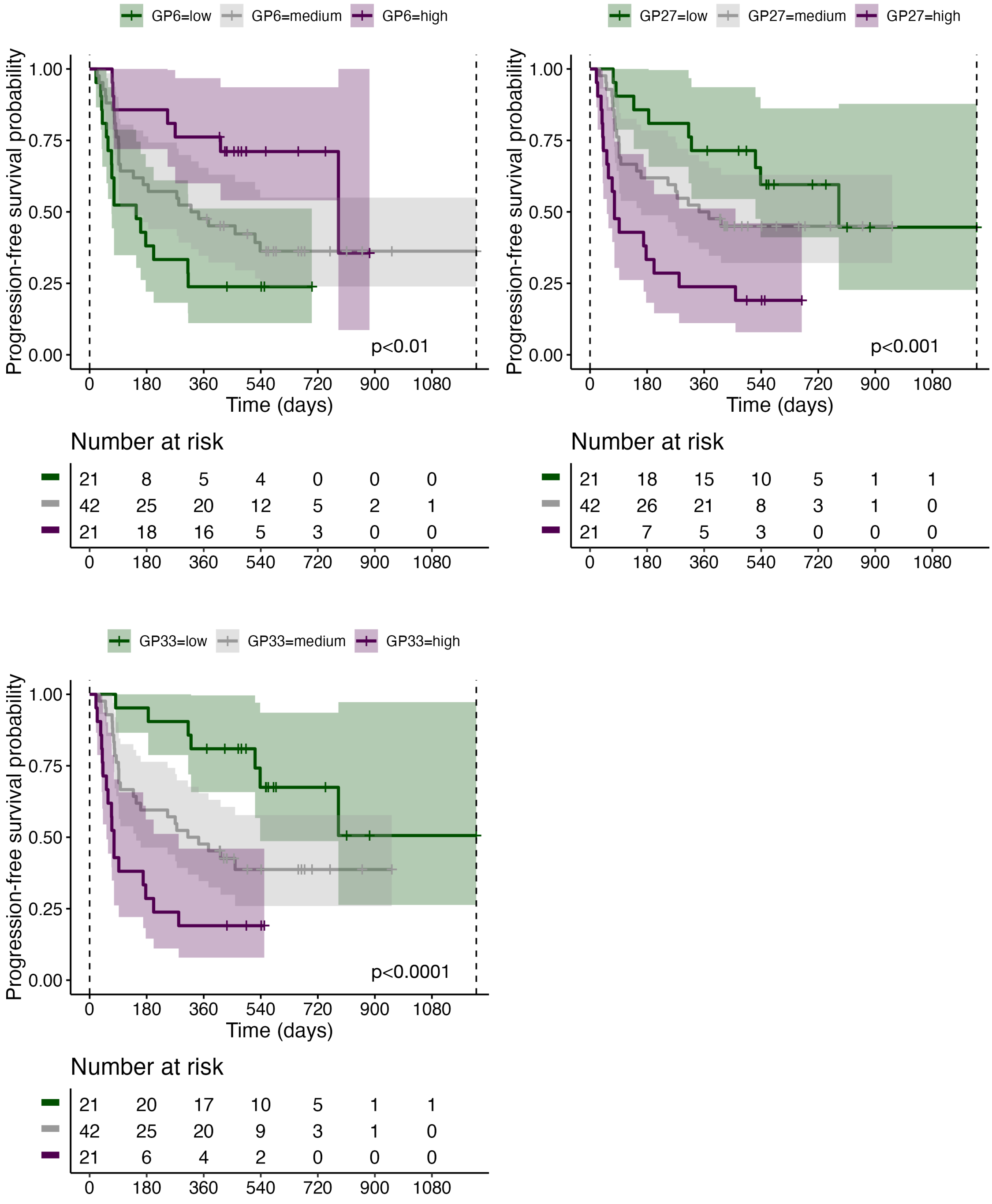

Supplementary Figure 5. **Kaplan-Meier progression-free survival curves in 88 patients with advanced melanoma (directly measured *N*-glycans).** Patients are divided in low (< 1st interquartile; dark green line), medium (> 1st interquartile and < 3rd interquartile; grey line), and high (> 3rd quartile; dark magenta line) according to their pre-treatment relative abundances of serum *N*-glycans. Dashed vertical lines indicate the intervals in which the *N*-glycan is prognostic according to a multivariate Cox proportional hazards regression analysis. Log-rank test *P* values are shown. Structures for the measured *N*-glycans: GP6: FA2[6]BG1, GP27: A3F1G3S2 (major structure)/A3G3S3 (low abundance), GP33: A3F1G3S3.

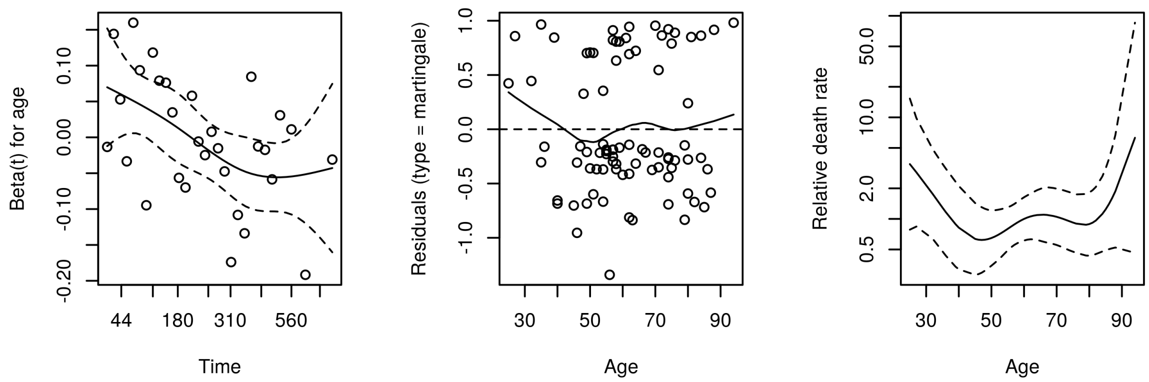

Supplementary Figure 6. Diagnostic plots for Cox proportional hazards regression model of survival time on sex, age at diagnosis of advanced melanoma, BMI, dichotomized LDH levels, and ECOG performance-status. **Left panel**: Index plots of dfbeta residuals for patients' age. The black line represents the LOWESS fit, and the dashed lines represent its 95% confidence intervals. **Middle panel**: Martingale residuals plot for patients' age. The black line represents the LOWESS fit. **Right panel**: Relative death rate as non-linear function of patients' age, as estimated using a Cox regression model with spline function. The curve is positioned so that patients aged 60, corresponding to the 50th percentile in our sample, have a relative death rate of 1. Dashed lines represent the 95% confidence intervals.

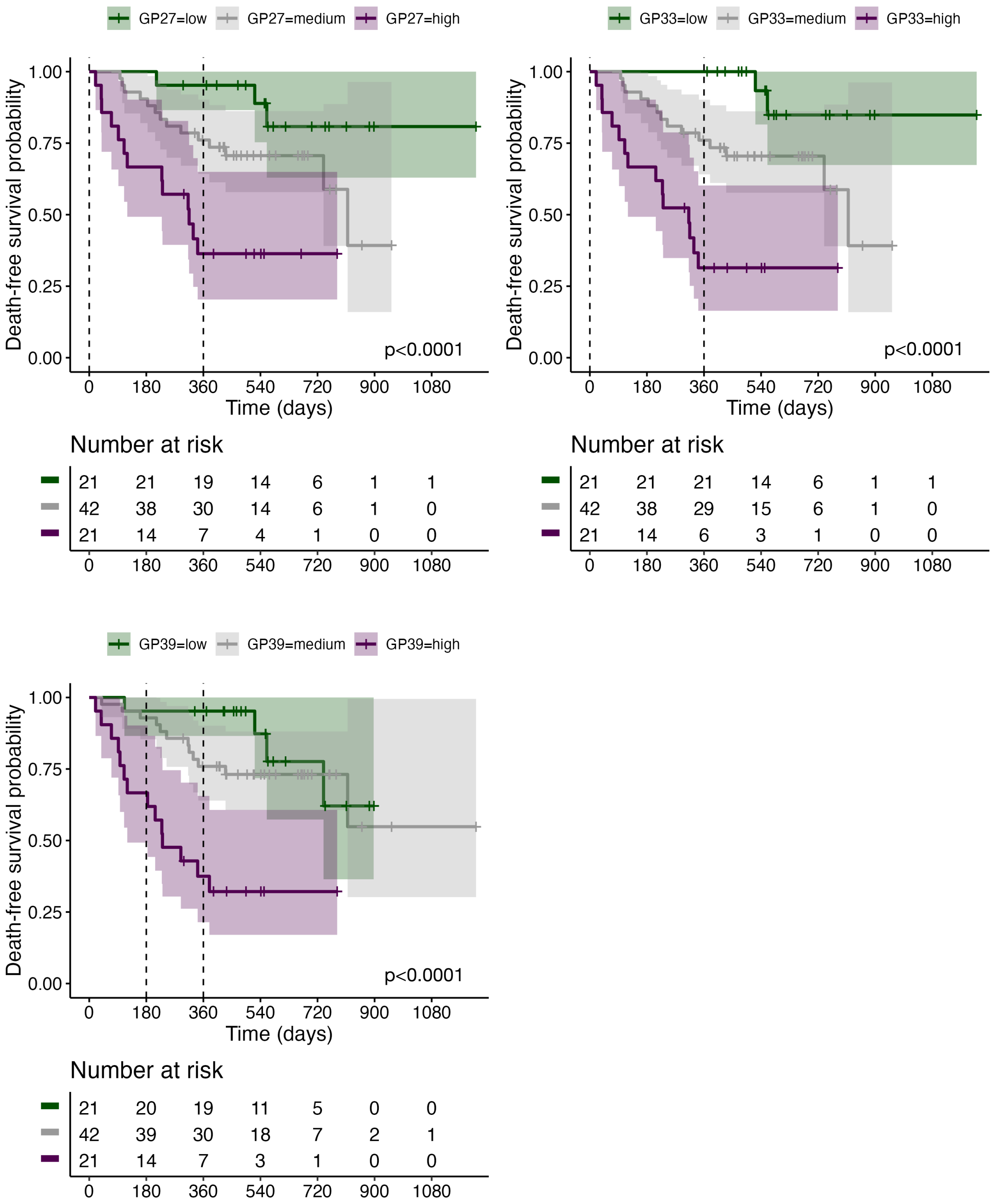

Supplementary Figure 7. **Kaplan-Meier overall survival curves in 88 patients with advanced melanoma (directly measured *N*-glycans).** Patients are divided in low (< 1st interquartile; dark green line), medium (> 1st interquartile and < 3rd interquartile; grey line), and high (> 3rd quartile; dark magenta line) according to their pre-treatment total serum *N*-glycan relative abundances. Dashed vertical lines indicate the intervals in which the *N*-glycan is prognostic according to a multivariate Cox proportional hazards regression analysis. Log-rank test *P* values are shown. Structures for the measured *N*-glycans: GP27: A3F1G3S2 (major structure)/A3G3S3 (low abundance), GP33: A3F1G3S3, GP39: A4F1G4S4 (major structure)/A4F2G4S4 (low abundance).
